# Response to ‘Evaluation of modelling study shows limits of COVID-19 importing risk simulations in sub-Saharan Africa’ (*Epidemiology and Infection* – HYG-LE-10513-May-20)

**DOI:** 10.1017/S0950268820001211

**Published:** 2020-06-10

**Authors:** Najmul Haider, Alexei Yavlinsky, Richard Kock

**Affiliations:** 1 The Royal Veterinary College, University of London, Hatfield, Hertfordshire AL9 7TA, UK; 2 Institute of Health Informatics, University College London, London, UK

To the Editor-in-Chief, We appreciate the letter by Miyachi *et al*. [1] about our paper, ‘Passengers’ destinations from China: low risk of Novel Coronavirus (2019-nCoV) transmission into Africa and South America’ [2]. In the letter, the authors state that they obtained 2417 COVID-19 cases reported by 40 countries in sub-Saharan Africa within the 30 days of the first case confirmed in Nigeria on 27 February. Of the 442 cases with international travel history, only one had travelled to China. We are encouraged by this finding and believe that it validates our modelling approach. The authors also point out that the model did not consider the risk of importing COVID-19 cases from other countries. We would like to point out that we submitted the final version of our manuscript to *Epidemiology and Infection* on 7 February 2020. At the time, virtually no instances of community transmission were being reported outside of China and thus there was no data available to reliably calculate the risk of case importation from other countries (please see WHO's situation Report-18 on Novel Coronavirus (2019-nCoV): https://www.who.int/docs/default-source/coronaviruse/situation-reports/20200207-sitrep-18-ncov.pdf?sfvrsn=fa644293_2).

It was also our hope that the public health measures that were being implemented in countries that were at high risk of importing COVID-19 cases would be sufficient to prevent further international spread of this disease, which unfortunately did not come to pass. At the time of manuscript submission, the possibility of pre/asymptomatic COVID-19 transmission was still a matter of debate within the scientific community, see, e.g. https://www.sciencemag.org/news/2020/02/paper-non-symptomatic-patient-transmitting-coronavirus-wrong.

However, we accept that the pre/asymptomatic transmission aspect of COVID-19 may have played a significant role in the collective failure to halt its spread and prevent it from becoming a global pandemic.
